# The association of QTc prolongation with cardiovascular events in cancer patients taking tyrosine kinase inhibitors (TKIs)

**DOI:** 10.1186/s40959-023-00178-x

**Published:** 2023-05-19

**Authors:** Ismail Ghafary, Chang-Kyung Kim, Eric Roth, Michael Lu, Erin M. Taub, Susan Lee, Ira Cohen, Zhongju Lu

**Affiliations:** 1 Department of Medicine, Stony Brook University, Stony Brook, NY 11794 USA; 2 Department of Physiology and Biophysics, Stony Brook University, Stony Brook, NY 11794 USA

**Keywords:** QTc prolongation, Tyrosine kinase inhibitors (TKIs), Cancer treatment, Cardiovascular events, Diabetes

## Abstract

**Objective:**

To investigate the association between stages of QTc prolongation and the risk of cardiac events among patients on TKIs.

**Methods:**

This was a retrospective cohort study performed at an academic tertiary care center of cancer patients who were taking TKIs or not taking TKIs. Patients with two recorded ECGs between January 1, 2009, and December 31, 2019, were selected from an electronic database. The QTc duration > 450ms was determined as prolonged. The association between QTc prolongation progression and events of cardiovascular disease were compared.

**Results:**

This study included a total of 451 patients with 41.2% of patients taking TKIs. During a median follow up period of 3.1 years, 49.5% subjects developed CVD and 5.4% subjects suffered cardiac death in patient using TKIs (n = 186); the corresponding rates are 64.2% and 1.2% for patients not on TKIs (n = 265), respectively. Among patient on TKIs, 4.8% of subjects developed stroke, 20.4% of subjects suffered from heart failure (HF) and 24.2% of subjects had myocardial infarction (MI); corresponding incidence are 6.8%, 26.8% and 30.6% in non-TKIs. When patients were regrouped to TKIs versus non-TKIs with and without diabetes, there was no significant difference in the incidence of cardiac events among all groups. Adjusted Cox proportional hazards models were applied to estimate hazard ratios (HRs) with 95% confidence intervals (CIs). There is a significant increased risk of HF events (HR, 95% CI: 2.12, 1.36–3.32) and MI events (HR, 95% CI: 1.78, 1.16–2.73) during the 1st visit. There are also trends for an increased incidence of cardiac adverse events associated with QTc prolongation among patient with QTc > 450ms, however the difference is not statistically significant. Increased cardiac adverse events in patients with QTc prolongation were reproduced during the 2nd visit and the incidence of heart failure was significantly associated with QTc prolongation(HR, 95% CI: 2.94, 1.73-5.0).

**Conclusion:**

There is a significant increased QTc prolongation in patients taking TKIs. QTc prolongation caused by TKIs is associated with an increased risk of cardiac events.

## Introduction

In response to stimulation with insulin, Tyrosine kinases (TKs) can be activated, which subsequently activate Phosphatidylinositol (PI) 3-kinases signaling (PI3K) [1]. PI3K signaling activity is important for cellular responses prompted by growth factors, whose elevation contributes to tumor genesis [2]. Over 90 TKs are known to be involved in tumor angiogenesis and malignant transformation [3]. The PI3K family members are amplified or mutated at high rates in an array of over 30 tumor types [1, 2]. Specific TK inhibitors have been established to interfere with TK enzymes that are critical to tumor growth and aberrantly activated in tumor cells by directly blocking/inhibiting the catalytic activity of TK as well as TK/PI3K signaling activity [4]. Such TKIs are nonselective and generally have potency against more than one receptor TK [4]. Since the first successful use of imatinib (Gleevec) in a chronic myeloid leukemia (CML) patient in 2001, over 50 TKIs have already been FDA approved for various cancers and more TKIs are currently in clinical trials [2],[5, 6].

Common adverse cardiac toxicities associated with TKIs include hypertension, left ventricular systolic dysfunction, congestive heart failure, and QT prolongation [7–9]. Lu et al. (2012) showed that TKIs are associated with QT prolongation in cardiomyocytes by PI3K signaling pathway inhibition and subsequent change in multiple ionic channels [10]. Due to the explosion of target therapy in cancer using TKIs and its related cardiovascular toxicity, modifications of risk factors for CVD including hyperlipidemia, HTN, diabetes, obesity have been suggested to prevent TKIs-induced cardiac toxicity in cancer patients [8]. However, our recent finding shows modification of cardiac risk factors has no significant effect on the TKIs-induced cardiac side effects [11].

PI3K regulates the action potential duration (APD) as well as cardiac contractility of individual myocytes as demonstrated by its altering of multiple ion currents in canine heart [10]. A previous study demonstrated that P13K signaling regulates both outward and inward currents that control APD [10]. PI3K inhibition caused reductions in the L-type calcium current (I_CaL_), the slowly (IKs) and rapidly (IKr) activating delayed rectifier potassium currents; in contrast, it triggered an increase in the persistent (late) sodium current (I_NaP_) [10]. A reduction of PI3K signaling in the heart caused reduced Ca^2+^ entry through the L-type calcium channel (LTCC) and subsequent reduction of left ventricular ejection fraction (LVEF) in the heart [12–14].

TKIs cause QTc prolongation and cardiovascular events [5, 7–9, 15]. We hypothesize that QTc interval can be a predictor of disease progression and thus a potential marker for monitoring cardiovascular events during TKI treatment. The association between QTc prolongation induced by TKIs and development of cardiovascular disease complications in patients taking TKIs will be evaluated.

## Patients and methods

### Study population

A retrospective cohort study was conducted using the database for patients registered at cancer center of Stony Brook University (SBU) approved by the Institutional Review Board (IRB number: 1703-039-836). This study’s participants retrospectively followed up from January 1, 2009 to December 31, 2019.

### Inclusion criteria

A series of 451 cancer patients with or without taking TKIs, with EKG recordings between January 1st, 2009, and December 31st, 2019, was identified by historical chart review.

Clinical physicians had prescribed TKIs for patients registered in SBU hospital for the treatment of cancer, and patients had taken TKIs for at least 1-month duration.

The definition of diabetes follows the diagnostic criteria set by the World Health Organization (WHO): fasting plasma glucose ≥ 7.0 mmol/L, or glycated hemoglobin (HbA1c) ≥ 6.5%, or 2 h plasma glucose ≥ 11.1 mmol/L, and undergoing treatment for diabetes, which included the use of oral hypoglycemic agents or insulin. Event of myocardial infarction was defined if with history of heart attack, s/p Cardiac stent(s), s/p coronary artery bypass grafting (CABG) or myocardial infarction (MI) were listed in the electric medical record (EMR). Event of stroke was defined for history of cerebral vascular disease, Transient ischemic attack (TIA), cerebral hemorrhagic or ischemia. Event of heart failure (HF) was defined if LVEF < 50% reported on transthoracic echocardiograph (TTE).

**Fig. 1:**
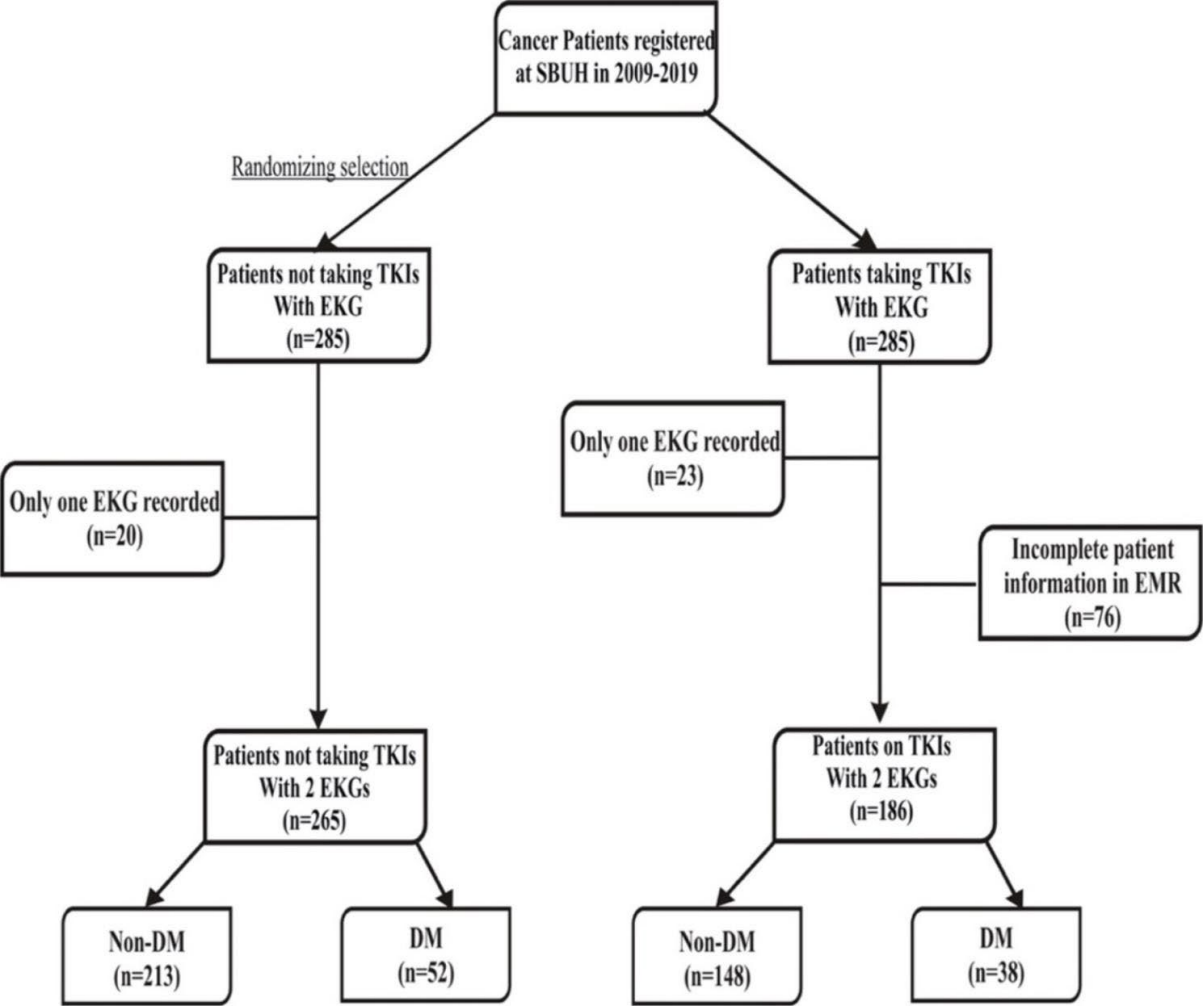
The Flow diagram of the study population

The flow diagram of the study population is shown in Fig. 1. According to the inclusion and exclusion criteria, patients were selected for data extraction in line with de-identified Health Insurance Portability and Accountability Act (HIPAA) compliance. A total of 186 patients taking TKIs were identified, and 285 patients on TKIs were randomized selected and identified as matched controls. Only 544 patients on TKIs had EKG recordings313 patients in total were selected according to our inclusion and exclusion criteria. A total of 99 observations were omitted from the analysis.

72 were omitted due to insufficient data on EMR, 4 were omitted due to MRN not being found and 13 were omitted due to no post EKG for those on TKI medication. 10 were omitted due to only 1 EKG available.

Patients with the following categories were excluded: (1) age less than 20, or older than 90; (2) BMI > 40; (3) on medication(s) that are known to prolong QTc intervals (for reference see website www.qtdrugs.org); (4) severe electrolyte abnormalities that prolong QT intervals.

### Key variables

The primary outcome was cardiovascular disease (CVD) including myocardial infarction (MI), heart failure (HF) and stroke. The secondary outcome including mortality and cardiac mortality. Event of cardiac mortality was defined as 2 or more days of hospitalization or death due to the International Classification of Diseases Tenth Revision (ICD-10) codes pertaining to CVD.

### Measurement of QTc interval from the 12-lead ECG

The standard 12-lead ECG tracing at 10 mm/mV amplitude and 25 mm/s paper speed was used. The QT interval was measured from the beginning of the earliest onset of the QRS complex to the end of the T wave. The end of the T wave was defined as the return of the descending limb to the TP baseline. QT intervals and the preceding RR intervals were measured on the resting ECG tracing in lead II. QTc was calculated using Bazett’s formula (QTc = QT/(RR)1/2 if HR is between 60 and 100 beat/min) or Fredericia formula (QTc = QT/(RR)1/3 if HR < 60 or > 100 beat/min) [16, 17]. Generally, due to the effect of endogenous hormonal modulation on the QTc, QTc interval > 450 ms in male and a QTc interval > 470 ms in female were considered abnormally prolonged [16, 17]. A prolonged QTc > 470 ms or an increase of > 30 ms from the baseline ECG has been shown with an increased risk of TdP [16, 18]. Thus, QTc > 450 has been used as the cutoff for QT prolongation based on the Common terminology criteria for adverse events (CTCAE) guidelines.

For patients who had multiple EKG’s and visits, after the initial visit the visit with the highest QTC was used. For those on TKIs, the EKG with highest QTc after drug initiation was used. For those who had no pre TKI EKG and the patient had multiple EKG’s, the first was taken as the initial. For those who didn’t have a TKI drug initiation date and the patient had multiple EKG’s with a TKI drug name given, then the first was taken as the initial.

### Statistical analysis

Baseline variables were summarized as mean ± standard error of mean (Mean ± SEM). The Student t-test (paired or non- paired) was used to test for differences between independent variables and the Chi-square test was used to test for differences between categorical variables. Data were collected, and subsequently mean or median and 95% confidence interval (CI) were determined. A probability value P < 0.05 was considered statistically significant.

The statistical analysis was conducted using SAS v9.4 (SAS Institute Inc., Cary, NC, USA). The data is paired on those subjects who are on TKI’s have a pre and post TKI EKG’s and on those subjects who aren’t on TKI’s but have 2 EKGs. Therefore, when making comparisons over time between continuous variables Wilcoxon Signed Rank tests were used. For categorical variables Mcnemar’s Test for paired data was used.

When making comparisons between those who are diabetic and non-diabetic, Chi-square test of independence or fisher exact tests were used for categorical variables. Fisher exact tests were used which help to adjust for small cell frequencies. For continuous variables that were not normally distributed a Wilcoxon rank sum test was used. A p-value of below 0.05 is considered to be statistically significant.

Continuous variables were expressed as mean ± standard deviation of mean, and frequency for descriptive statistics.

Kaplan -Meier curves are given for adverse events, showing the survival function. It gives the probability of survival based on time from initial visit and death.

Cox hazard regression was used for all-cause mortality, no other adverse events were modeled due to lack of time to event dates.

Logistic regression was used to model the probability of adverse events, currently only univariate models are given. We can discuss multivariate models, but they cannot be done with stroke and cardiac mortality due to low event rate.

## Results

### Baseline characteristics

Among the 451 studied subjects, 53.7% were men with a mean age of 62.7 years old, 90.5% are non-Hispanic, 56.1% are obese with a mean BMI of 27.3, and the mean HbA1C 6.5. Among participants, 48.1% have hypertension, 23.1% have dyslipidemia, and 31.3% have alcohol use. Among all studied subjects, the mean LVEF was 58.3% for the 1st visit and 54.2% for the 2nd visit, respectively; and the mean QTc was 438.8 for the 1st visit and 474.3 for the 2nd visit, respectively.

In Summary, for patients on TKIs (n = 265), the mean QTc was 441.0 for the 1st visit and 473.9 for the 2nd visit, respectively. For patients not on TKIs (n = 186), the mean QTc was 436.7 for the 1st visit and 474.8 for the 2nd visit, respectively. In both TKIs and Non-TKIs, there is a significant increase of QTc intervals on the 2nd visit when compared to that in the 1st visit suggesting QTc prolongation during the follow-up. As shown in the Table 1, status of diabetes does not affect the QTc significantly in both on TKIs and Non-TKIs groups.

**Table 1:** Patient’s Baseline Characterization

n = 451	Patients not on TKI’s (n = 265)		P-Value	Patients on TKI’s (n = 186)		P-Value
	Non-DM(n = 213)	DM(n = 52)		Non-DM(n = 148)	DM(n = 38)	
Age (years)	62.92 ± 13.22	67.18 ± 9.72	0.0161	58.67 ± 12.95	61.81 ± 12.46	0.1930
Time between Visits (years)	3.08 ± 3.22	2.63 ± 3.21	0.3606	3.59 ± 3.35	3.23 ± 3.08	0.5271
Gender, n (%)						
Male	116 (54.72)	30 (57.69)	0.6990	70 (48.28)	26 (68.42)	0.0269
Female	96 (45.28)	22 (42.31)		75 (51.72)	12 (31.58)	
Race, n (%)						
Caucasian	186 (88.15)	42 (80.77)	0.2134	125 (86.81)	24 (64.86)	0.0192
African American	12 (5.69)	5 (9.62)		8 (5.56)	6 (16.22)	
Asian	4 (1.90)	0 (0.0)		4 (2.78)	3 (8.11)	
Other	9 (4.27)	5 (9.62)		7 (4.86)	4 (10.81)	
Ethnicity, n (%)						
Non-Hispanic	196 (95.15)	48 (94.12)	0.7258	133 (93.66)	31 (91.18)	0.7035
Hispanic	10 (4.85)	3 (5.88)		9 (6.34)	3 (8.82)	
Obesity, n (%)						
BMI < 25	85 (41.26)	22 (42.31)	0.9837	61 (44.53)	12 (31.58)	0.1442
BMI 25–30	66 (32.04)	16 (30.77)		30 (21.90)	14 (36.84)	
BMI ≥ 30	55 (26.70)	14 (26.92)		46 (33.58)	12 (31.58)	
BMI	26.62 ± 6.71	26.86 ± 6.47	0.8125	27.25 ± 7.19	28.64 ± 7.50	0.3109
HbA1c	5.51 ± 0.53	7.40 ± 1.19	< 0.0001	5.41 ± 0.57	7.60 ± 1.28	< 0.0001
Hypertension, n(%)						
No	99 (46.48)	23 (4.23)	0.7706	93 (62.84)	19 (50.0)	0.1492
Yes	114 (53.52)	29 (55.77)		55 (37.16)	19 (50.0)	
Dyslipidemia, n(%)						
No	164 (77.0)	33 (63.46)	0.0451	123 (83.11)	27 (71.05)	0.0934
Yes	49 (23.0)	19 (36.54)		25 (16.89)	11 (28.95)	
Alcohol Use, n (%)						
None	144 (67.61)	36 (69.23)	0.8831	103 (69.59)	27 (71.05)	0.7482
Monthly	28 (13.15)	6 (11.54)		14 (9.46)	2 (5.26)	
Weekly	18 (8.45)	6 (11.54)		16 (10.81)	5 (13.16)	
Daily	9 (4.23)	1 (1.92)		1 (0.68)	1 (2.63)	
Abuse	14 (6.57)	3 (5.77)		14 (9.46)	3 (7.89)	
Heart rate (BPM)	80.59 ± 20.36	84.67 ± 19.58	0.1849	83.26 ± 20.48	81.08 ± 20.89	0.5671
QTc (ms), 1st Visit	436.31 ± 30.66	437.10 ± 35.06	0.8817	436.70 ± 34.48	445.21 ± 36.82	0.2035
LVEF (%), 1st Visit	57.64 ± 12.85	57.44 ± 13.77	0.9329	59.13 ± 12.22	58.72 ± 10.83	0.8568
QT_C_ (ms), 2nd Visit	471.89 ± 44.62	477.71 ± 53.25	0.4686	473.46 ± 53.99	474.29 ± 40.34	0.9167
LVEF(%), 2nd Visit	55.11 ± 15.21	45.50 ± 20.51	0.6263	60.23 ± 14.12	56.00 ± 27.07	0.8137

Similarly, the mean LVEF was 58.9% for the 1st visit and 58.1% for the 2nd visit in all patients on TKIs, respectively. The mean LVEF was 57.5% for the 1st visit and 50.3% for the 2nd visit in all patients not on TKIs, respectively. There is a significant reduction of LVEF in Patients with DM during the follow up, the mean LVEF was 57.4% for the 1st visit and 45.5% for the 2nd visit (Table 1).

### Cardiac events Induced by TKIs in patients with QTc prolongation

During a mean follow-up of 3.13 years, among all the patients with or without TKIs, we observed that 174 cases were observed to be death related; 13 cases were attributed to CVD; 262 subjects developed cardiovascular disease.

For all patients on TKIs (n = 186), 77 subjects suffered all-cause mortality, 10 subjects suffered cardiac mortality, and 92 subjects developed cardiovascular disease. A total of 38 subjects developed heart failure, 9 subjects suffered a stroke, and 45 subjects suffered a myocardial infarction (MI).

For all Non-TKI patients (n = 265), the results show that 97 subjects suffered all-cause mortality, 3 subjects suffered cardiac mortality, and 170 subjects developed cardiovascular disease. A total of 71 subjects developed heart failure, 18 subjects suffered a stroke, and 81 subjects suffered a myocardial infarction (MI).

In comparison to the randomized control patients who were not taking TKIs, there is no significant difference in the accumulated occurrence and prevalence of cardiac events, mortality and cardiac mortality in patients taking TKIs (Table 2).

**Table 2:** Cardiac Events Induced by TKIs in Patients with QTc Prolongation

Incidences of events	Non-TKI (n = 265)	TKI (n = 186)	QT_C_ <450, n (%)			QT_C_ 450–479, n (%)			QT_C_ 480–499, n(%)			QT_C_ ≥500, n (%)			QT_C_ ≥450 all, n (%)		
			1st	2nd	P Value	1st	2nd	P Value	1st	2nd	P Value	1st	2nd	P Value	1st	2nd	P Value
**MI, n (%)**																	
No	184 (69.43)	141 (75.8)	102 (81.60)	59 (85.51)	0.003	27 (64.29)	33 (67.35)	0.759	9 (75.0)	18 (78.26)	0.827	3 (42.86)	31 (68.89)	0.178	92 (63.89)	195 (66.10)	0.647
Yes	81 (30.57)	45 (24.19)	23 (18.40)	10 (14.49)	0.003	15 (35.71)	16 (32.65)	0.759	3 (25.0)	5 (21.74)	0.827	4 (57.14)	14 (31.11)	0.178	52 (36.11)	100 (33.90)	0.647
**HF, n (%)**																	
No	194 (73.21)	148 (79.57)	105 (84.0)	59 (85.51)	0.015	30 (71.43)	39 (79.59)	0.365	9 (75.0)	18 (78.26)	0.827	4 (57.14)	32 (71.11)	0.456	95 (65.97)	206 (69.83)	0.414
Yes	71 (26.79)	18 (20.43)	20 (16.0)	10 (14.49)	0.015	12 (28.57)	10 (20.41)	0.365	3 (25.0)	5 (21.74)	0.827	3 (42.86)	13 (28.89)	0.456	49 (34.03)	89 (30.17)	0.414
**Stroke, n(%)**																	
No	247 (93.21)	177 (95.16)	119 (95.20)	68 (98.55)	0.204	40 (95.24)	45 (91.84)	0.515	12 (100.0)	23 (100.0)	NA	6 (85.71)	41 (91.11)	0.652	135 (93.75)	276 (93.56)	0.939
Yes	18 (6.79)	9 (4.84)	6 (4.80)	1 (1.45)	0.204	2 (4.76)	4 (8.16)	0.515	0 (0.0)	0 (0.0)	NA	1 (14.29)	4 (8.89)	0.652	9 (6.25)	19 (6.44)	0.939
**All-Cause Mortality**, n(%)																	
No	168 (63.40)	109 (58.60)	78 (62.40)	51 (73.91)	0.218	21 (50.0)	28 (57.14)	0.496	6 (50.0)	9 (39.13)	0.537	4 (57.14)	21 (46.67)	0.606	83 (57.64)	167 (56.61)	0.838
Yes	97 (36.60)	77 (41.4)	47 (37.60)	18 (26.09)	0.218	21 (50.0)	21 (42.86)	0.496	6 (50.0)	14 (60.87)	0.537	3 (42.86)	24 * (53.33)	0.6058	61 (42.36)	128 (43.39)	0.838
**Cardiac Mortality**, n(%)																	
No	262 (98.87)	117 (95.16)	119 (95.20)	68 (98.55)	0.062	39 (92.86)	42 (85.71)	0.277	12 (100.0)	22 (95.65)	1.000	6 (85.71)	44 (97.78)	0.1226	139 (96.53)	284 (96.27)	0.893
Yes	3 (1.13)	10 (5.38)	6 (4.80)	1 (1.45)	0.0619	3 (7.14)	7 (14.29)	0.2774	0 (0.0)	1 (4.35)	1.0000	1 (14.29)	1 (2.22)	0.1226	5 (3.47)	11 (3.73)	0.893

**Fig. 2:**
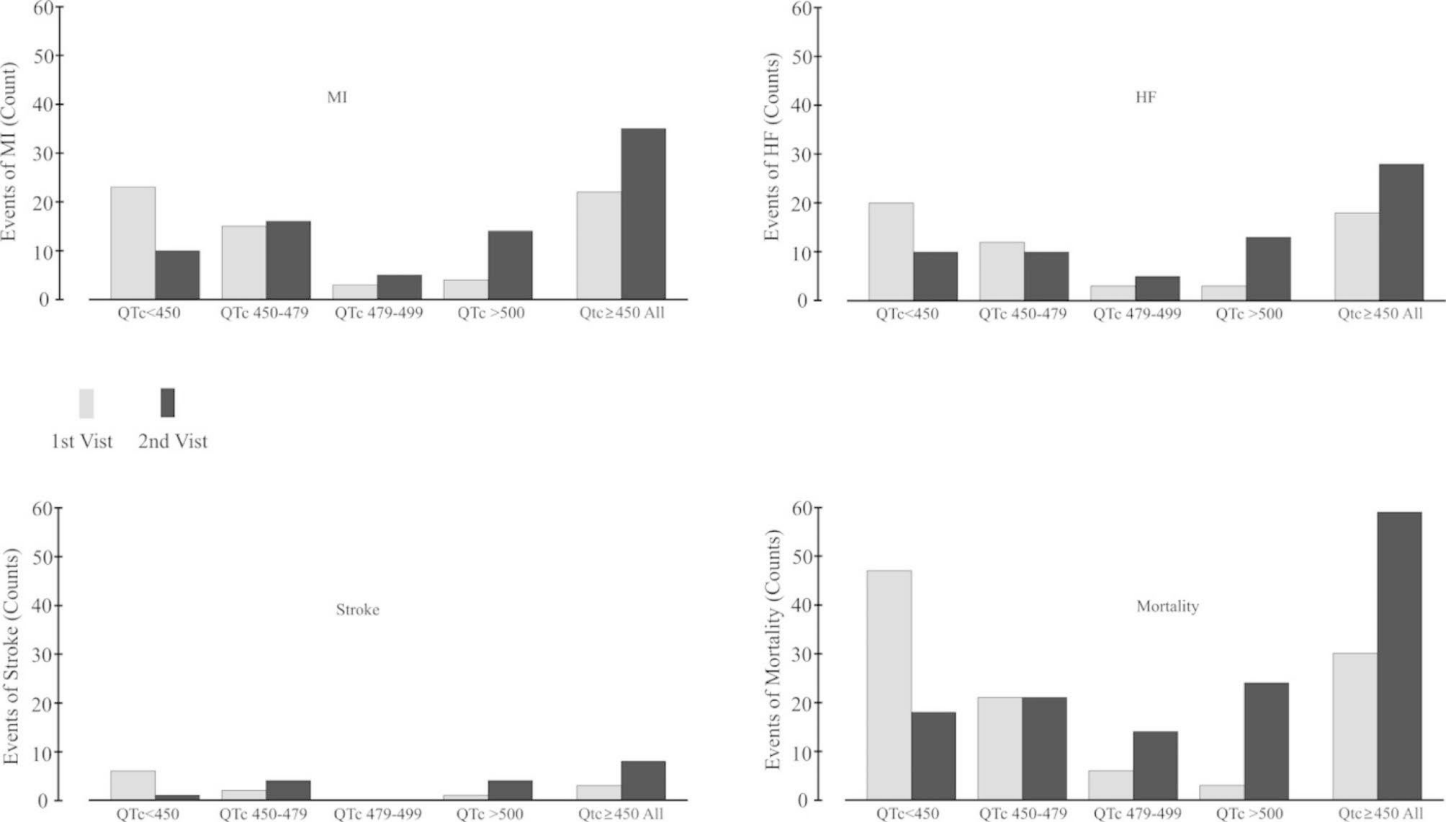
Cardiac Events Induced by TKIs in Patients with QTc Prolongation

The cardiac events in TKIs patients were further analyses when divided into groups of QTc < 450ms, QTc 450-479s, QTc 480-500ms, QTc > 500ms. The cumulated cardiac events in each groups were compared between the 1st visit and 2nd visit. As illustrated in Fig. 2, the events of MI were the highest cardiac events induced by TKIs, followed by Heart Failure and stroke. The cumulated cardiac events were higher in the 2nd visit compared to that in the 1st visit. There is a trend that cardiac events increased with the increase of QTc prolongation, but the difference is not statistically significant.

### Adverse events in patients on TKIs with DM

We have reported previously that patients on TKIs have prolonged QTc intervals when they are diabetic [11]. Thus, the association between occurrence of cardiac events and DM among patients on TKIs was further examined.

For patients on TKIs with diabetes (n = 38), 16 subjects suffered all-cause mortality, 2 subjects suffered cardiac mortality, 20 subjects developed cardiovascular disease. Among the CVD events, 6 subjects developed heart failure, 4 subjects suffered a stroke, and 10 subjects suffered a myocardial infarction (MI). For patients on TKIs without diabetes (n = 148), 61 subjects suffered all-cause mortality, 8 subjects suffered cardiac mortality, and 72 subjects developed cardiovascular disease. A total of 32 subjects developed heart failure, 5 subjects suffered a stroke, and 35 subjects suffered a MI, respectively.

In the control group, 52 patients are diabetic. 18 subjects suffered all-cause mortality, 0 subjects suffered cardiac mortality, and 35 subjects developed cardiovascular disease, respectively. A total of 15 subjects developed heart failure, 4 subjects suffered a stroke, and 16 subjects suffered a myocardial infarction (MI). There are 213 patients who are non-diabetic not on TKIs. 79 subjects suffered all-cause mortality, 3 subjects suffered cardiac mortality, and 135 subjects developed cardiovascular disease. A total of 56 subjects developed heart failure, 14 subjects suffered stroke, and 65 subjects suffered a MI, respectively (Table 3).

**Table 3:** Adverse cardiac Events in Patients on TKIs with and without DM

Adverse Event	Myocardial Infarction, n (%)			Heart Failure, n (%)			Stroke, n (%)			Cardiac Mortality, n (%)			All-Cause Mortality, n (%)		
	Yes	No	P Value	Yes	No	P Value	Yes	N o	P Value	Yes	No	P Value	Yes	No	P Value
**Non-TKI** **(n = 265)**															
Non DM (n = 213)	65 (30.52)	148 (69.48)	0.9717	56 (26.29)	157 (73.71)	0.7092	14 (6.57)	199 (93.43)	0.7608	3 (1.41)	210 (98.59)	0.3894	79 (37.09)	134 (62.91)	0.7399
DM (n = 52)	16 (30.77)	36 (69.23)	0.9717	15 (28.85)	37 (71.15)	0.7092	4 (7.69)	48 (92.31)	0.7608	0 (0.0)	52 (100.0)	0.3894	18 (34.62)	34 (65.38)	0.7399
**TKIs** **(n = 186)**															
Non DM (n = 148)	35 (23.65)	113 (76.35)	0.7320	32 (21.62)	116 (78.38)	0.4264	5 (3.38)	143 (96.62)	0.0863	8 (5.41)	140 (94.59)	0.9723	61 (41.22)	87 (58.78)	0.9209
DM (n = 38)	10 (26.32)	28 (73.68)	0.7320	6 (15.79)	32 (84.21)	0.4264	4 (10.53)	34 (89.47)	0.0863	2 (5.26)	36 (94.74)	0.9723	16 (42.11)	22 (57.89)	0.9209

### Multivariate analysis of cardiac adverse events in patient on TKIs

As illustrated in Table 4, there is no significant difference on cardiac adverse events in diabetic patients when compared to those in non-diabetic patients.

**Table 4:** Cox regression analysis for association between stages of QTc prolongation and adverse events

QTc Categories	All-cause mortality		Cardiac Mortality		HF incidences		MI incidences		Stroke incidences	
	HR (95% CI)	P Value	HR (95% CI)	P Value	HR (95% CI)	P Value	HR (95% CI)	P Value	HR (95% CI)	P Value
**Diabetes**										
Yes VS No	0.96 (0.60,1.54)	0.862	0.72 (0.16,3.32)	0.677	0.94 (0.55,1.63)	0.836	1.06 (0.64,1.77)	0.822	0.70 (0.31,1,59)	0.392
**On TKIs**										
Yes VS No	1.22 (0.83,1.80)	0.304	4.96 (1.35,18.29)	0.016	0.70 (0.45,1.10)	0.121	0.73 (0.47,1.11)	0.138	1.76 (0.74,4.15)	0.200
**QTc Prolongation at 1st Visit**										
≥ 450 VS < 450	1.26 (0.84,1.89)	0.259	1.35 (0.43,4.18)	0.609	2.12 (1.36,3.32)	0.001	1.78 (1.16,2.73)	0.009	1.07 (0.47,2.44)	0.872
480–499 vs. 450–479	1.56 (0.67,3.63)	0.630	0.001 (0.001,999)	0.943	0.26 (0.08,0.81)	0.031	1.31 (0.55,3.12)	0.864	2.28 (0.51,10.19)	0.396
≥ 500 vs. 450–479	1.56 (0.54,4.51)	0.681	4.62 (0.71,30.12)	0.931	0.94 (0.32,2.79)	0.304	2.03 (0.70,5.89)	0.296	1.27 (0.14,11.61)	0.873
**QTc Prolongation at 2nd Visit**										
≥ 450 VS < 450	1.83 (1.21,2.77)	0.004	2.98 (0.65,13.62)	0.159	2.94 (1.73,5.00)	0.001	2.56 (1.58,4.17)	0.001	1.27 (0.54,2.98))	0.577
480–499 vs. 450–479	1.98 (1.00,3.92)	0.124	0.39 (0.05,3.23)	0.739	1.06 (0.47,2.39)	0.224	0.98 (0.47,2.04)	0.466	0.81 (0.16,3.93)	0.572
≥ 500 vs. 450–479	1.42 (0.85,2.36)	0.968	0.32 (0.07,1.53)	0.449	2.89 (1.67,5.01)	0.003	1.60 (0.95,2.70)	0.08	1.53 (0.57,4.12)	0.314

**Table 5:** Cardiac Events by TKIs Drug Name

TKIs	QTc (ms)		MI	HF	Stroke	Mortality	Cardiac Mortality
	Mean ± SEM	≥ 450,n(%)	n (%)	n (%)	n (%)	n (%)	N (%)
Alectinib (N = 1)	555	1 (100)	1 (100)	1 (100)	0 (0.0)	0 (0.0)	0 (0.0)
Axitinib (N = 2)	426.5 ± 22.5	0 (0.0)	0 (0.0)	0 (0.0)	0 (0.0)	1 (50)	0 (0.0)
Carbozantinib (N = 4)	453.5 ± 18.8	2 (50.0)	2 (50.0)	0 (0.0)	0 (0.0)	1 (25.0)	0 (0.0)
Crizotinib (N = 6)	450.0 ± 34.2	2 (33.3)	2(33.3)	0 (0.0)	0 (0.0)	2 (33.3)	1 (16.7)
Dasatinib (N = 6)	527.8 ± 90.3	6 (100)	2(33.3)	1(16.67)	1(16.6)	1 (16.67)	1 (16.67)
Erlotinib (N = 12)	469.6 ± 48.8	8 (66.67)	2(16.6)	2(16.67)	0 (0.0)	5 (41.67)	1 (8.33)
Ibrutinib (N = 20)	459.3 ± 45.9	12 (60.0)	5(25.0)	5 (25.0)	2(10.0)	7 (35.0)	0 (0.0)
Imatinib (N = 27)	460.6 ± 38.4	17 (63.0)	4(14.8)	9(33.3)	0 (0.0)	12 (44.4)	3 (11.1)
Lapatinib (N = 4)	465.0 ± 27.4	2 (50)	1 (25.0)	1 (25.0)	0 (0.0)	2 (50.0)	0 (0.0)
Lenvatinib (N = 2)	507 ± 32.0	2 (100)	1 (50.0)	0 (0.0)	1 (50)	1 (50)	0 (0.0)
Midostaurin (N = 3)	528.3 ± 80.1	3 (100)	0 (0.0)	1(33.33)	0 (0.0)	1 (33.33)	0 (0.0)
Nilotinib (N = 8)	492.6 ± 40.2	6 (75.0)	6(75.0)	2 (25.0)	2 (25.0)	2 (25.0)	0 (0.0)
Pazopanib (N = 12)	456.9 ± 26.5	7 (58.3)	4(33.30)	5 (41.7)	2 (16.7)	7 (58.3)	2 (16.7)
Ponatinib (N = 1)	548	1 (100)	0 (0.0)	0 (0.0)	0 (0.0)	1 (100)	0 (0.0)
Regorafenib (N = 1)	624	1 (100)	0 (0.0)	0 (0.0)	0 (0.0)	1 (100)	0 (0.0)
Ruxolitinib (N = 6)	494.7 ± 61.7	4 (66.7)	0 (0.0)	1(16.7)	0 (0.0)	3 (50.0)	2 (33.3)
Sorafenib (N = 32)	492.2 ± 50.0	24 (75.0)	10(31.3)	4 (12.5)	1(3.1)	15 (46.9)	0 (0.0)
Sunitinib (N = 11)	457.5 ± 22.2	6 (54.5)	2 (18.2)	3(27.3)	0 (0.0)	2 (18.2)	0 (0.0)
Tofacitinib (N = 1)	417	0 (0.0)	0 (0.0)	0 (0.0)	0 (0.0)	0 (0.0)	0 (0.0)
Trametinib (N = 2)	412.5 ± 12.5	0 (0.0)	0 (0.0)	0 (0.0)	0 (0.0)	2 (100)	0 (0.0)

When compared with control patients (non-TKIs), using TKIs can cause a greater risk for cardiac mortality (HR, 95% CI: 4.96, 1.35–18.29). There is a trend for higher risk of stroke incidence (HR, 95% CI: 1.76, 0.74–4.15) and all-cause mortality (HR, 95% CI: 1.22, 0.83–1.80), which is not statistically significant.

When comparing adverse events among all QTc categories during the 1st visit, the risk of HF, MI, stroke, cardiac mortality, and all-cause mortality is observed to be higher in patient with QTc prolongation (QTc > 450ms). There is a significant increased risk of HF events (HR, 95% CI: 2.12, 1.36–3.32) and MI events (HR, 95% CI: 1.78, 1.16–2.73) during the 1st visit. There are trends for an increased incidence of cardiac adverse events between group QTc 480–499 versus group QTC 450–479 and group QTc > 500 versus group QTC 480–499, however the difference is not statistically significant. Similarly increased cardiac adverse events in patients with QTc prolongation were seen during the 2nd visit. There is a significant difference in the incidence of HF events (HR, 95% CI: 2.94, 1.73-5.0), MI events (HR, 95% CI: 2.56, 1.58–4.17) and all-cause mortality (HR, 95% CI: 1.83, 1.21–2.77), respectively. A very significant increased risk in the incidence of HF events was noticed when QTc > 500ms (HR, 95% CI: 2.89, 1.67-5.0), (Table 4)

### Cardiac events association with QTc prolongation among different TKIs

The Mean QTc intervals and corresponding cardiac events, mortality, and cardiac mortality in different TKIs are shown in Table 5. There is a trend that increased QTc prolongation are associated with increased cardiac events among different TKIs. Patients with QTc > 500ms were identified as high-risk with known poor outcomes for cardiovascular disease [19]. TKIs with long QTc > 500 ms including Alectinib (QTc 555 ms), Dasatinib (QTc 527.8 ms), Levatinib (QTc 507 ms), Midostaurin ( QTc 528.3 ms), Ponatinib ( QTc 528 ms) and Regorafenib ( QTc 624 ms) show cardiac events with no significant difference compared to other TKIs with QTc < 500ms.

## Discussion

The present study confirmed that there is increased QTc prolongation in patients taking TKIs. This is a novel study to demonstrate that QTc prolongation caused by TKIs is associated with increased risk of cardiac events. Previously we have demonstrated additional QTc prolongation in patients taking TKIs if patients are diabetic [11]. However, our current study shows no significant changes of cardiac events if patients are diabetic and taking TKIs.

### Cardiovascular side effects caused by TKIs

Previous studies have shown vascular events (including cerebrovascular, cardiovascular, and peripheral vascular events) when cancer patients received TKIs [20]. The development and clinical utility of target therapy using TKIs has been limited due to its cardiovascular toxicities including left ventricular dysfunction, QT prolongation and arrhythmia, ischemia and myocardial infarction, congestive heart failure, and hypertension [8, 9, 21]. Among the TKIs, vandetanib, nilotinib and sunitinib have been reported for their QT prolonging effects and potential cardiac mortality clinically [22]. Sunitinib has been reported to demonstrate cardiovascular-related adverse events in a dose-dependent manner within the clinic [23]. A recent study has shown that sorafenib, palbociclib, ruxolitinib, and eriotinib demonstrated significantly higher absolute QTc intervals and prevalence of severe QTc prolongation [11]. A large scale met-analysis of 45 randomized controlled trials (a total of 20,027 patients) associated with nine FDA-approved VEGFR-TKIs demonstrates Lenvatinib, followed by vandetanib, cabozantinib, vxitinib, pazopanib, sorafenib, sunitinib, regorafenib and nintedanib had the greatest probability of producing all grades cardiovascular incident and hypertension [24]. QTc prolongation, LV dysfunction, and hypertension can be managed effectively through reliable methods of carefully monitoring patients and clinical management, which requires experience of both a cardiologist and an oncologist, in a developing subspecialty called cardio-oncology. The need for effective pharmacovigilance and continuous re-assessment of their risk/benefit in clinic is clear given the data on morbidity and mortality associated with TKIs. Clinicians should be mindful of cardiovascular toxicity and perform regular cardiovascular monitoring [25]. If not adequately managed, these cardiovascular effects of TKIs significantly increase the morbidity and mortality and decrease the quality of life in cancer patients.

### Molecular mechanism of cardiovascular toxicities of TKIs

Although there is consensus that exposure to TKIs confers risks for cardiovascular diseases in patents, the underlying mechanisms are still unclear [8, 22]. Various factors have been postulated to play different roles in the cardiovascular toxicities of TKIs, such as endothelial damage and atherosclerosis [26], as well as hypertension effect [27]. Nilotinib has been shown to inhibit human microvascular endothelial cell proliferation and survival, both in the presence and absence of VEGF. Nilotinib is also related to an increase of markers of apoptosis including caspase-3 and − 7, which determines damage that partly resumes the one known to be caused by VEGF-inhibiting TKIs [26]. Moreover, in endothelial cells of in vitro models, nilotinib also shown to cause an increased expression of VCAM-1, ICAM-1, and E-selectin as pro-atherogenic surface molecules [28].

The molecular basis of QTc prolongation in diabetes has been demonstrated in various diabetic models to be a results of reduced PI3K signaling activity in the heart, followed by prolongation of APD due to altering of cardiac ion currents [10, 14, 29]. It is known that Tyrosine kinase (TK) can be activated upon insulin stimulation, which subsequently activate PI3K [1]. Diabetes is associated with reduced TK-PI3K signaling, which affect the APD of individual myocytes and subsequently the QT interval, by modulating several ion currents in canine heart [12]. Previous experiments suggest that the cause of the QT prolongation in the diabetic mice can be attributed to decreased insulin/PI3K signaling [13]. Patients with diabetes might exhibit reduced TK/PI3K signaling in the heart due to insulin resistance or insufficiency, which leads to multiple cardiac ion current/channels irregularities and subsequent QT prolongation. Likewise, the decrease of LVEF in diabetes may be attributed to a decreased PI3K signaling in the heart since reduced Ca^2+^ entry through the L-type calcium channel (LTCC), which might exhibit a negative effect on cardiac contractility, has been found in type 1 and type 2 diabetic mice [13, 14]. Although the mechanism of cardiovascular toxicity by TKIs has not been elucidated, the reduced TK-PI3K signaling, and subsequent alternation of multiple ion channels may shed light on the molecular mechanism.

### The limitation of this study

This is a retrospective study designed to identify the relationship between the QTc prolongation and cardiovascular side effects. A long-term cohort study possessing randomized controls would help elucidate the cause of cardiac side effects and QTc prolongation. The study design should further consider factors including heterogeneity of the study group, dose dependence of TKIs and the need for a larger sample size of patients. A prospective study could help validate the relationship between TKIs-induced cardiac toxicity and the control/modification of common CVD risk factors. Many potential confounding variables for QTc prolongation should be controlled in future studies.

## Conclusions

Use of TKIs is associated with a significantly increased risk of QTc prolongation and cardiovascular events in cancer patients. TKIs causes a significant prolongation of QTc prolongation, with further prolongation of QTc in diabetic patients. There is a significant increased risk of HF events and MI events by TKIs and the incidence of heart failure is significantly increased among patient with QTc > 450ms. The greater QTc prolongation caused by TKIs is associated with more cardiovascular events, suggesting that QTc interval (or changes) can be a useful marker for monitoring and predicting cardiovascular side effects caused by TKIs.

## Data Availability

Dataset is available on secured website at Stony Brook University.
